# Harnessing natural compounds and nanotechnology for miRNA-based osteosarcoma therapy

**DOI:** 10.1039/d5ra06010a

**Published:** 2026-02-09

**Authors:** Abdullah AlAqel, Gharieb S. El-Sayyad, Shaza H. Aly, Ahmed E. Elesawy, Osama A. Mohammed, Hend H. Mohamed, Walaa A. El-Dakroury, Maha Abdelsalam, Ahmed S. Doghish

**Affiliations:** a Department of Biomedical Engineering, College of Engineering, International University of Kuwait (IUK) Ardiya Kuwait; b Drug Microbiology Lab., Drug Radiation Research Department, National Center for Radiation Research and Technology (NCRRT), Egyptian Atomic Energy Authority (EAEA) Cairo Egypt Gharieb.S.Elsayyad@eaea.org.eg; c Department of Pharmacognosy, Faculty of Pharmacy, Badr University in Cairo (BUC) Cairo 11829 Egypt; d Department of Biochemistry, Faculty of Pharmacy, Badr University in Cairo (BUC) Badr City Cairo 11829 Egypt; e Department of Pharmacology, College of Medicine, University of Bisha Bisha 61922 Saudi Arabia; f School of Biotechnology, Badr University in Cairo (BUC) Badr City Cairo 11829 Egypt; g Department of Pharmaceutics and Industrial Pharmacy, Faculty of Pharmacy, Badr University in Cairo (BUC) Badr City Cairo 11829 Egypt; h Department of Clinical Pathology, Faculty of Medicine, Mansoura University Mansoura 35516 Egypt; i Biochemistry and Molecular Biology Department, Faculty of Pharmacy (Boys), Al-Azhar University Nasr City Cairo 11231 Egypt ahmed_doghish@azhar.edu.eg; j Clinical Pathology Department, Armed Force College of Medicine Cairo Egypt

## Abstract

Osteosarcoma remains one of the most challenging malignancies due to its aggressive nature, metastatic potential, and resistance to conventional therapies. Recent advances have underscored the pivotal role of microRNAs (miRNAs) in the regulation of key oncogenic and tumor suppressor pathways involved in osteosarcoma progression, including PI3K/AKT, Wnt/β-catenin, and TGF-β signaling. The modulation of miRNAs offers a promising therapeutic avenue, but effective delivery systems are essential to realize their full potential. Natural compounds derived from plants, such as flavonoids, resveratrol, quercetin, and epigallocatechin-3-gallate, have demonstrated notable capacity to modulate miRNA expression, inducing apoptosis, inhibiting proliferation, and reducing metastasis with fewer adverse effects compared to traditional chemotherapy. These bioactive molecules possess intrinsic anti-inflammatory, antioxidant, and osteogenic properties, which, when combined with miRNA regulation, can synergistically impede osteosarcoma progression. Nanotechnology-based delivery systems, including multilayered nanoparticles, magnetic nanostructures, and biodegradable nanocarriers, have emerged as effective platforms to overcome these obstacles. These nanocarriers can be engineered for targeted, controlled, and sustained release of therapeutic agents, enhancing accumulation at tumor sites while minimizing systemic toxicity. Additionally, functionalization with targeting ligands such as folic acid or antibodies further improves specificity towards osteosarcoma cells. This review emphasizes the potential of combining natural compounds with nanotechnology to develop innovative, targeted, and effective therapies for osteosarcoma. It highlights the opportunities for future research to optimize delivery systems, elucidate mechanisms of action, and establish clinical applicability. By harnessing the biological benefits of natural agents and the precision of nanomedicine, these approaches hold significant promise for improving therapeutic outcomes and reducing adverse effects in osteosarcoma treatment.

## Abstract 2

This review emphasizes the potential of combining natural compounds with nanotechnology to develop innovative, targeted, and effective therapies for osteosarcoma.

## Graphical abstract

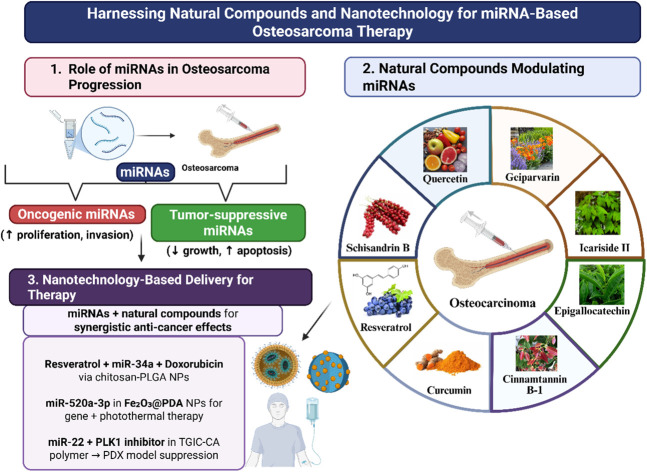

## Introduction

Osteosarcoma is a highly aggressive malignant tumor originating from primitive mesenchymal cells that form osteoid tissue, predominantly affecting children and young adults. It is characterized by rapid progression, a high propensity for metastasis, particularly to the lungs, and resistance to conventional chemotherapy and radiotherapy, which significantly complicates treatment strategies.^1,2^ Despite advances in surgical techniques and chemotherapeutic regimens, the overall survival rate for osteosarcoma patients has plateaued over recent decades, highlighting the urgent need for innovative therapeutic approaches.^3^

In recent years, the molecular understanding of osteosarcoma has deepened, revealing critical roles for microRNAs (miRNAs), small, non-coding RNA molecules that regulate gene expression post-transcriptionally, in tumor initiation, growth, metastasis, and resistance to therapy.^4^ Aberrant expression of specific miRNAs has been linked with oncogenic processes in osteosarcoma; for instance, some miRNAs promote proliferation and metastasis, while others function as tumor suppressors. Consequently, modulating miRNA levels presents a promising strategy for targeted therapy, aiming to restore normal regulation of oncogenic pathways.^5^

Natural products derived from plants and other organisms have long served as sources of therapeutic agents due to their structural diversity, multi-target effects, and generally favorable safety profiles.^6–8^ Compounds such as flavonoids (*e.g.*, icariin, quercetin, resveratrol), epigallocatechin-3-gallate (EGCG), and other bioactive phytochemicals have demonstrated significant anti-tumor activities in various cancers, including osteosarcoma.^9,10^ These bioactive substances can influence multiple molecular pathways, including the regulation of miRNA expression, leading to apoptosis induction, inhibition of metastasis, and chemosensitization.^9–11^

However, despite their therapeutic potential, the clinical application of natural compounds in cancer treatment faces considerable challenges. Issues such as poor bioavailability, instability under physiological conditions, and non-specific distribution limit their effectiveness.^12^ To address these limitations, nanotechnology-based delivery systems have been developed, offering improved stability, targeted delivery, and controlled release of therapeutic agents. Nanoparticles can be engineered to carry miRNAs, natural bioactive compounds, or both, enabling synergistic anti-tumor effects while minimizing off-target toxicity. Surface modifications with targeting ligands, such as folic acid or specific antibodies, further enhance the selectivity of nanocarriers towards osteosarcoma cells.^13–15^

Integrating natural compounds with nanotechnology to deliver miRNAs constitutes a promising strategy that exploits the biological activity of phytochemicals and the precision of nanomedicine. This approach aims to modulate critical signaling pathways involved in osteosarcoma progression, suppress tumor growth, and prevent metastasis more effectively than traditional therapies alone. As research advances, such strategies can pave the way for more effective, less toxic, and personalized treatments for osteosarcoma patients.^16,17^

This review article explores current developments in the use of natural compounds and nanotechnology for miRNA-based osteosarcoma therapy, emphasizing their mechanisms of action, recent innovations, and future prospects to improve clinical outcomes.

## Role of miRNAs in osteosarcoma development

A deep investigation of signaling pathways in osteosarcoma formation is crucial for enhancing patient survival rates.^18^ MiRNAs have a varied regulatory role in osteosarcoma formation. While certain miRNAs can act as oncogenes, others can reduce tumors by regulating target gene expression.^18^ The regulation of osteosarcoma by miRNAs is linked to signaling system activity and protein molecule expression levels.^19^ Studying molecular pathways, including PTEN/AKT, PI3K/AKT, Wnt/β-catenin, NF-κB, and proteins like IRF2, P21, BCL9L, and VEPH1, may aid in osteosarcoma research.

## miRNAs boost osteosarcoma proliferation

Many studies indicate that miRNAs influence osteosarcoma development. MiRNAs play a role in promoting osteosarcoma proliferation through unique molecular processes.^20^ Understanding the regulation mechanisms of miRNAs in osteosarcoma is crucial for therapeutic benefit.

P21 is an important metabolic regulator in cells; it also stimulates tumor growth and has the potential to be an anticancer target molecule.^21^ There is a strong correlation between the clinical stage of osteosarcoma and the expression of miR-95-3p. Through the TGF-beta/CDKN1A/p21/cyclin D1 pathway, miR-95-3p overexpression stimulates osteosarcoma cell growth and prevents apoptosis.^22^ P21 has a strong inhibitory effect on CDKs, especially CDK2 and CDK4. Reducing cell mass while increasing proliferation and transformation through overexpression of the cyclin-CDK complex speeds up cell cycle checkpoint bypass. Inhibition of cyclin-CDK *via* the P21waf1/cip1 pathway is essential for checkpoint control.^23^ In response to DNA damage, the tumor suppressor protein p53 meticulously regulates the transcriptional level of CDKN1A/p21 expression.^23,24^ Further, miR-95-3p overexpression promotes tumor cell migration and proliferation in cultured cells and growth in xenograft animal models by adversely regulating p21 by targeting the 3′-UTR.^25^

An essential intracellular signaling pathway that controls cell cycle progression is PI3K/AKT. Osteosarcoma is one of many cancers that initiate this signaling pathway.^26,27^ By blocking apoptosis, this signaling pathway encourages cell proliferation, survival, and EMT.^28^ In esophageal, hepatocellular, and colon squamous cell carcinomas, miR-23 b-3p has been proven to be carcinogenic. The miR-23 b-3p promotes osteosarcoma by targeting the VEPH1/PI3K/AKT signaling pathway.^29^ miR-23 b-3p targets SIX1 to trigger apoptosis and inhibit osteosarcoma cell growth and invasion.^30^ A study found that miR-18a-5p inhibits IRF2 expression, promoting OS cell invasion and migration.^31^ However, LncRNA FER1L4 inhibits OS progression by downregulating miR-18a-5p production and blocking the PI3K/AKT signaling pathway.^32^

One of the most frequently altered genes in human tumors is PTEN, a well-established tumor suppressor gene.^33^ The lipid phosphatase PTEN dephosphorylates the 3′ position of phosphatidylinositol-34,5-trisphosphate (PIP3), which is produced by the proto-oncogenic PI3K, activating the PI3K pathway.

AKT kinase is a crucial target for cancer treatment due to its function in cell survival, proliferation, angiogenesis, and anabolism.^34^ A major cancer treatment target. miR-21, -216, and -524 can boost osteosarcoma cell growth by targeting PTEN and activating PI3K/Akt signaling.^20,35–37^ Alterations in the PI3K/AKT signaling pathway may be necessary to prevent osteosarcoma progression, as it is uncontrolled in most localized diseases and all advanced diseases.^20^

Activating the WNT/β-catenin signaling pathway has been retained throughout evolution and is crucial for tissue development and homeostasis *in vivo*.^38^ In human cancers, such as cervical, nasopharyngeal, and stomach tumors, Wnt/β-catenin signaling is significantly active. Numerous techniques targeting Wnt/β-catenin signaling have been developed as cancer therapeutics due to the contribution of genetic and epigenetic dysregulation to human cancer.^39^ According to recent studies, the Wnt/β-catenin pathway is used by baicalein, alantolactone, and schisandrin B to decrease osteosarcoma.^40–42^ Some miRNAs have been found to influence the Wnt/β-catenin signaling pathway and promote osteosarcoma.^20^ Osteosarcoma can be advanced by targeting this signaling pathway with miR-377-3p and miR-22-3p.^43,44^ According to research, miR-214-3p has the potential to promote osteosarcoma oncogenesis by activating the Wnt/β-catenin/LEF1 signaling pathway through targeting DKK3. This process is not entirely facilitated by cantharidin, though. Cantharidin has the potential to target the miR-214-3p/DKK3/β-catenin signaling pathways, making it a potential therapy for osteosarcoma^45^ (Table 1).

**Table 1:** miRNAs boost osteosarcoma proliferation

miRNAs	Signaling pathways	Target genes	Effects	Experimental model
miR-95-3p	P21	Caspase-3, caspase-9, and Bax	Stimulates osteosarcoma cell growth and prevents apoptosis^22^	*In vitro*
miR-23 b-3p	PI3K/AKT	VEPH1	Promotes osteosarcoma growth^29^	*In vitro*
miR-18a-5p		SOCS5	Promotes OS cell invasion and migration^32^	*In vitro*
miR-21	PTEN/PI3K/AKT	PTEN	Boosts osteosarcoma cell growth^35–37^	*In vitro*
miR-216				
miR-524				
miR-377-3p	Wnt/β-catenin	*CUL1*	Promotes the progress of osteosarcoma^43–45^	*In vitro*
miR-22-3p		TCF7L2		
miR-214-3p		DKK3		

## miRNAs inhibit osteosarcoma proliferation

Furthermore, numerous investigations have discovered that osteosarcoma under-expresses a substantial number of miRNAs.^20^ These miRNAs can be increased to treat osteosarcoma by lowering tumor proliferation and blocking tumor growth. Understanding the targets and signaling cascades of these miRNAs may lead to therapeutic targets.

The gene astrocyte elevated gene-1 (AEG-1) is involved in osteosarcoma, tumor growth, and metastasis regulation, and is associated with carcinogenic signaling pathways such as Wnt and NF-κB.^46^ As an example, the actions of miR-342-3p and miR-448 in inhibiting osteosarcoma growth involve directing AEG-1 to obstruct Wnt and NF-κB signaling pathways.^47,48^ The NF-κB pathway regulates gene expression, promotes proliferation, prevents apoptosis, and regulates responsiveness to ageing agents and cytokines, contributing to osteosarcoma pathogenesis.^49^ An instance of this is how miR-29a inhibits the SOCS1/NF-κB signaling pathway, which in turn decreases osteosarcoma cell proliferation.^50^ The NF-κB pathway was responsible for osteosarcoma cell death and growth inhibition due to miR-155 downregulation.^51^

Evidence suggests that anti-osteosarcoma effects can be achieved by inhibiting the PI3K/Akt and Wnt/β-catenin pathways. To inhibit the proliferation of osteosarcoma cells, miR-564 inhibits Akt.^52^ Furthermore, blocking the Wnt/β-catenin signaling pathway is how miR-152 prevents osteosarcoma cell proliferation^53^ (Table 2).

**Table 2:** miRNAs inhibit osteosarcoma proliferation

miRNAs	Signaling pathways	Target genes	Effects	Experimental model
miR-342-3p	Wnt and NF-κB	AEG-1	Inhibits osteosarcoma growth^47,48^	*In vitro*
miR-448				
miR-29a	SOCS1/NF-κB	DNMT3B	Decreases osteosarcoma cell proliferation^50^	*In vitro*
miR-564	PI3K/Akt	Akt	Prevents osteosarcoma cell proliferation^52,53^	*In vitro*
miR-152	Wnt/β-catenin	DKK1		*In vitro*

## Natural products and miRNA regulation in osteosarcoma

Natural products are bioactive compounds originating from biogenic sources, including phytogenic, microbial, or mineral origins, which exhibit a broad spectrum of pharmacological activities such as anti-inflammatory, antioxidative, and antineoplastic properties.^6–8^ Natural products are essential for the progression of therapeutics owing to their structural variety, multi-target efficacy, and historical effectiveness in the treatment of diseases.^54–58^ The advancement of cancer therapies has been significantly influenced by natural compounds, which utilise various molecular mechanisms and exhibit fewer side effects compared to conventional pharmaceuticals.^7,59^ Osteosarcoma, an exceptionally aggressive bone malignancy, presents significant treatment challenges due to medication resistance and metastatic capability.^60^ Recent studies emphasise the significance of miRNAs in the regulation of critical oncogenic pathways,^61,62^ whereas natural products have surfaced as potential modulators of these miRNAs to improve therapeutic results.^61,63^ The following section presents a summary of current research about the mechanisms by which natural products interact with miRNAs to modulate apoptosis, chemosensitivity, autophagy, and metastasis in osteosarcoma (Table 3).

**Table 3:** Natural products and their approaches in regulating miRNA expression in osteosarcoma

Natural product	Class	Source	Osteosarcoma cancer cell lines/model	Concentration/dose	Target(s) and function(s)
Icariside II (ICS II)	Flavonoid	*Epimedium brevicornu*	K7M2 and 143B cells	3, 6, 12 µM	↓ Migration and invasion
					Inhibit phosphoglycerate kinase 1 (PGK1)
					↓ miR-194/215 expression
			K7M2 cells were rinsed twice with PBS, and 1 × 10^6^ cells in 200 µl PBS were administered *via* tail vein injection into the mice	12.5, 25, and 50 mg kg^−1^ day^−1^	Suppressed lung metastasis in Balb/C mice
					↓ miR-194/215 expression^64^
Resveratrol	Phenolic compound	Grapes	U2OS, MG63 cells	5, 10 and 20 µM	↑ miR-139-5p
					↑ Caspase-3
					↑ Apoptosis
					↓ NOTCH1 (ref. 65)
Curcumin	Polyphenolic compound	Turmeric (*Curcuma longa*)	MG-63 and HOS OS cells CCK-8 assay	2.5, 5 and 10 µM	↓ miR-21
					↑ RECK
					↑ Apoptosis
					↓ Cell viability
					Suppressed Wnt/β-catenin signaling^66^
Cinnamtannin B-1 (CTB-1)	Proanthocyanidin	*Cinnamomum zeylanicum* and *Laurus nobilis*	OS cells (HOS, MG63, 143B, U2OS) and normal osteoblasts (hFOB 1.19)	10, 20 and 40 µM	↓ Proliferation
					↓ Migration/invasion
					Suppressed Ki67 (proliferation marker)
					↑ miR-1281
			BALB/c nude mice with HOS xenografts treated with CTB-1	20 mg kg^−1^	↓ Tumor volume/weight
					↑ miR-1281
					↓ PPIF expression^67^
Geiparvarin	Coumarin derivative	Australian Willow (*Geijera parviflora*)	HOS and 143B	0.25, 0.5, 1, 2 µg mL^−1^	↓ Cell migration and invasion
					↓ miR-3912-3p
					↑ ANGPTL4 (angiogenin-like protein 4)
			BALB/c nude mice were injected with 143B cells to assess lung metastasis	5 mg kg^−1^	Reduced OS lung metastasis without causing toxicity^68,69^
			MG-63, HOS cells	—	↑ Apoptosis
					↓ Cell proliferation 7 & metastasis
					↓ Survivin expression
					↓ COX2 expression
Schisandrin B (Sch B)	Lignan	Fructus Schizandrae	SaOS2, U2OS and MG63 clls	20, 40 and 80 µM	↑ Apoptosis
					↓ Cell viability, migration, and invasion
					↓ miR-708-5p
					↑ circ_0009112 (ref. 70) inactivation of the PI3K/AKT pathway
Quercetin	Flavonoid	Various fruits and vegetables	143B	5, 10 µM	↑ miR-217
					↓ Cell proliferation
					↓ KRAS
					↑ Cytotoxic effects of cisplatin^71^
Epigallocatechin-3-gallate (EGCG)	Polyphenolic		MG63 and U2OS OS cells	0.025–0.2 g L^−1^	↓ Cell proliferation
					↑ miR-126
					Overexpression of miR-126 augmented the inhibitory effects of EGCG on cell proliferation^72^

**Fig. 1:**
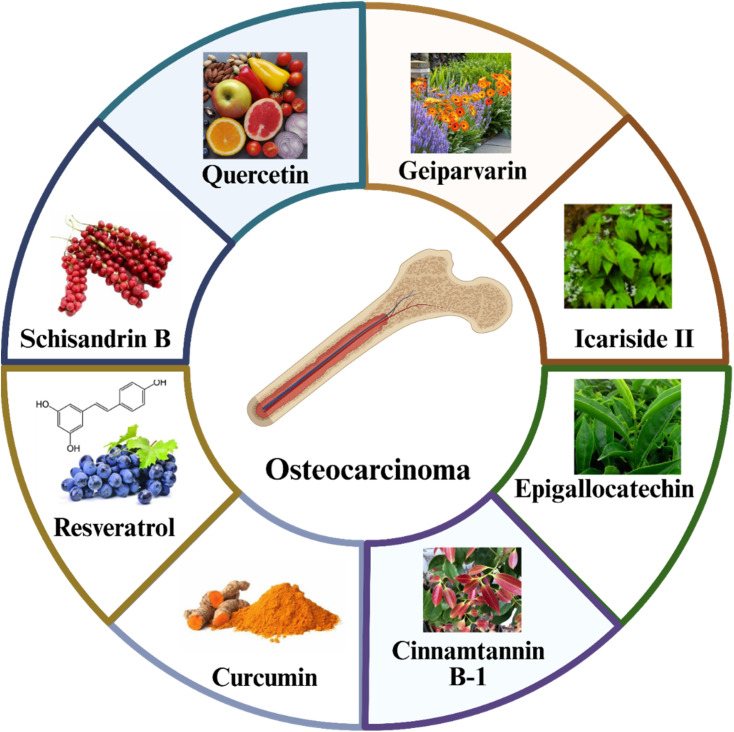
The natural compounds that have potential activity towards osteosarcoma cells.

**Fig. 2:**
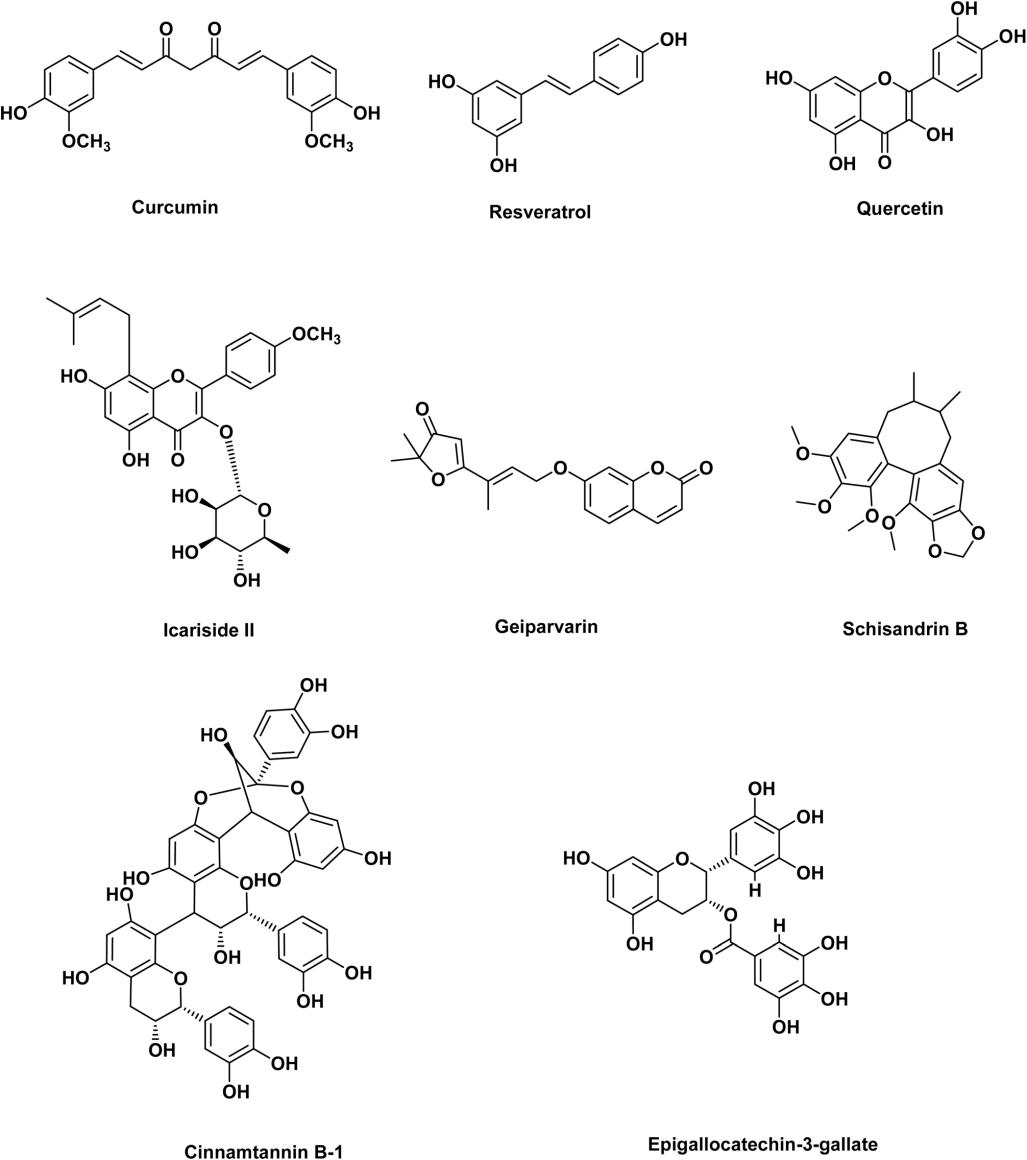
Chemical structures of different natural compounds with potential activity towards osteosarcoma cells.

The potential of Icariside II (ICS II), a bioactive flavonoid from *Epimedium brevicornu,* was investigated to inhibit epithelial–mesenchymal transition (EMT) and metastasis in osteosarcoma by targeting the oncogenic miR-194/215 cluster through modulation of phosphoglycerate kinase 1 (PGK1) (Fig. 1 and 2). ICS II directly binds to the ADP-binding pocket of PGK1, competitively inhibiting its enzymatic activity. PGK1 inhibition disrupts glycolysis and suppresses miR-194/215-driven EMT, reducing metastatic potential. The *in vitro* study showed that ICS II significantly reduced the migration and invasion of K7M2 cells, which are a mouse metastatic osteosarcoma cell line, and osteosarcoma 143B cells. The *in vivo* study showed that ICS II treatment suppressed lung metastasis in mouse models, correlating with decreased PGK1 activity and miR-194/215 expression.^64^

Resveratrol is a phenolic and phytoalexin compound produced by various plants in response to damage or infections and is most abundant in different berry types and grapes. It has been demonstrated to possess anticancer properties in both cell culture and animal models of various cancers^62,73^ (Fig. 1 and 2). Zou *et al.* revealed that resveratrol suppresses OS progression by inhibiting the canonical Wnt/β-catenin pathway, where the mRNA and protein expression levels of β-catenin and c-Myc were markedly downregulated, leading to reduced proliferation and increased apoptosis in MG-63 cells.^74^ Resveratrol markedly enhanced apoptosis in OS cell lines, such as U2OS and MG63, through the activation of caspase-3 in a dose-dependent manner. Levels of miR-139-5p were diminished in osteosarcoma cells relative to normal osteoblasts, and resveratrol administration recovered its expression. Resveratrol upregulated miR-139-5p, leading to NOTCH1 downregulation and subsequent inhibition of OS cell proliferation and survival. NOTCH1 knockout mimicked resveratrol's pro-apoptotic effects, confirming its role in the pathway.^65^ This study supports the development of resveratrol-based therapies or miRNA-targeting strategies for OS treatment.

Curcumin is a naturally occurring polyphenolic compound isolated from the rhizomes of *Curcuma longa*. A member of the Zingiberaceae family, it is a spice frequently utilized for food preservation and medicinal purposes. Curcumin may perform numerous activities due to its wide variety of functional properties, which include anti-cancer capabilities.^55,62^ It inhibits osteosarcoma progression by reducing miR-21 expression, alleviating its inhibition of RECK (reversion-inducing cysteine-rich protein with kazal motifs), a tumor suppressor. Increased RECK levels inhibit the Wnt/β-catenin pathway, downregulating pro-metastatic proteins as β-catenin and GSK-3β, it also reduces osteosarcoma cell proliferation, invasion, and survival. The *in vitro* study revealed that curcumin treatment in MG-63 and HOS OS cells suppressed miR-21, upregulated RECK, and reduced Wnt/β-catenin signaling, leading to apoptosis and inhibited migration. Western blot analysis revealed the upregulation of pro-apoptotic Bax and downregulation of anti-apoptotic Bcl-2. It concluded that inhibition of the miR-21/RECK axis reduces EMT markers and MMP-9 activity, limiting metastasis.^66^ Li *et al.* showed that curcumin, a natural polyphenol, suppresses OS cell growth and invasion *via* modulating the Notch-1 signaling pathway. The study utilizing MG-63 and HOS OS cell lines demonstrated that curcumin causes G2/M phase cell cycle arrest and inhibits invasion by downregulating Notch-1 and its downstream effectors, including matrix metallopeptidase 2 (MMP-2), and matrix metallopeptidase 9 (MMP-9).^75^

Cinnamtannin B-1 (CTB-1) is a naturally occurring proanthocyanidin present in *Cinnamomum zeylanicum* and *Laurus nobilis*. CTB-1 dose-dependently inhibited the proliferation of OS cell lines, such as HOS, MG63, 143B, and U2OS, but spared normal osteoblasts (hFOB 1.19). It reduced migration/invasion of OS cells through the Transwell assay and suppressed Ki67, a proliferation marker. miRNA sequencing revealed that miR-1281 is significantly upregulated in CTB-1-treated HOS cells. Moreover, PPIF (Peptidylprolyl Isomerase F), a mitochondrial protein associated with apoptosis resistance, was confirmed as a direct target of miR-1281 by a dual-luciferase reporter test. CTB-1 decreased PPIF expression in OS cells, facilitated by miR-1281. In a xenograft mouse model, CTB-1 reduced tumor volume/weight and increased miR-1281 while decreasing PPIF expression^67^ (Fig. 1 and 2).

Geiparvarin is a coumarin derivative found in the leaves of the Australian Willow. It inhibited the viability of OS cell lines (HOS and 143B) in a dose-dependent manner. It reduced OS cell migration and invasion *in vitro*. Mechanistically, geiparvarin downregulates miR-3912-3p expression in OS cells. By inhibiting miR-3912-3p, geiparvarin upregulates ANGPTL4 (angiogenin-like protein 4) expression, leading to reduced metastatic potential of OS cells. In a xenograft mouse model, geiparvarin reduced OS lung metastasis without causing toxicity.^68^ Another study revealed that geiparvarin significantly inhibited MG-63 and HOS cells' proliferation, invasion, and metastasis, and mouse xenograft models as well. It also induced apoptosis in OS cells by activating caspase-3 and reducing survivin expression. Moreover, geiparvarin treatment decreased COX2 expression, a key mediator of inflammation and tumor progression. Reduced COX2 levels correlated with diminished angiogenesis *via* VEGF suppression and inhibition of lung metastasis.^69^

Schisandrin B (Sch B) is a lignan derived from Fructus Schizandrae, a well-known Chinese herb. Sch B suppresses cell viability, migration, and invasion while promoting apoptosis in osteosarcoma OS cell lines. circ_0009112 is upregulated in OS cells, while miR-708-5p is downregulated. Sch B reverses this imbalance, downregulating circ_0009112 and upregulating miR-708-5p in a dose-dependent manner. That resulted in the inactivation of the PI3K/AKT pathway.^70^

Quercetin is a naturally occurring flavonoid prevalent in various fruits and vegetables. A dosage of 5 µM quercetin markedly increased cisplatin sensitivity in the OS cell line 143B, but elevated levels more than 10 µM directly suppressed the cells' proliferation. Quercetin enhanced the expression of miR-217, which directly targets and inhibits KRAS, an oncogene associated with chemoresistance. KRAS downregulation at both mRNA and protein levels impaired pro-survival signaling, hence augmenting the cytotoxic effects of cisplatin.^71^

Epigallocatechin-3-gallate (EGCG) at doses ranging between 0.025–0.2 g L^−1^ suppressed the proliferation of MG63 and U2OS OS cells in a dose- and time-dependent manner. At 0.05 g L^−1^, EGCG's inhibitory effects were comparable to 20 µM cisplatin. The overexpression of miR-126 in U2OS cells augmented the inhibitory effects of EGCG on cell proliferation by increasing G1 phase arrest and apoptosis. Combining EGCG with miR-126. Overexpression synergistically enhanced apoptosis and inhibited proliferation.^72^

**Fig. 3:**
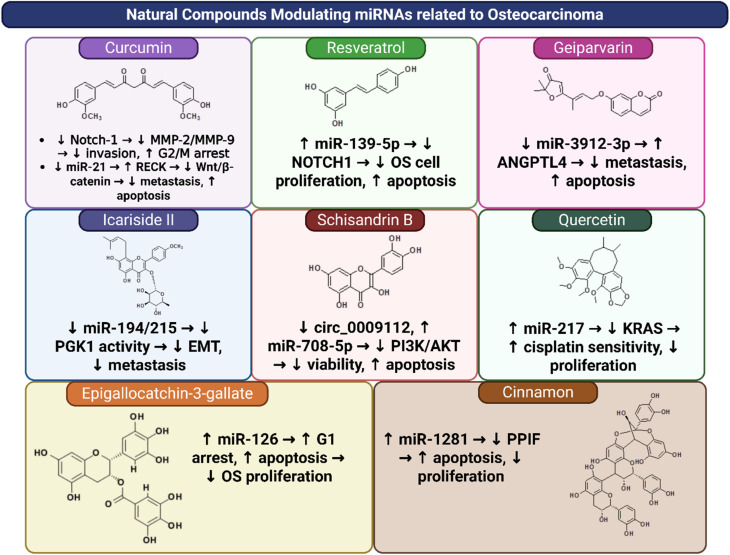
List of natural compounds targeting specific miRNA-pathway axes.

Natural products modulate miRNAs to disrupt osteosarcoma progression through apoptosis induction, autophagy regulation, and chemo-sensitization. While promising, challenges such as standardization, bioavailability, and clinical validation remain. Future research should focus on nanoparticle delivery systems and combination therapies to harness the full potential of miRNA-targeting natural products in OS treatment. The chemical structures of different natural compounds with potential activity towards osteosarcoma cells are illustrated in Fig. 2 and 3.

Based on the above-mentioned studies, Icariside II (ICS II), Cinnamtannin B-1 (CTB-1), and geiparvarin represent the most promising compounds for inhibiting metastasis. They have been investigated in both *in vitro* cell lines (K7M2, 143B, HOS) and *in vivo* mouse models, where they prevented lung metastasis and tumor growth. In contrast, resveratrol and curcumin predominantly demonstrate efficacy in apoptosis induction through the miR-139-5p/NOTCH1 and miR-21/RECK pathways, although they lack direct *in vivo* metastasis data, which hinders their comparative potential for aggressive osteosarcoma. Quercetin and EGCG exhibit chemosensitizing properties *via* augmenting cisplatin through miR-217/KRAS, suggesting their greater effectiveness in combination therapies rather than monotherapy.

## Nanoparticles based treatment

The application of nanoparticles is widespread in treating several cancer types, including osteosarcoma. Nanoparticles offer distinct advantages, rendering them a formidable asset in contemporary oncology.^12^ Their diminutive size facilitates superior infiltration into tumor tissues, augmenting medication accumulation at the tumor location while reducing off-target effects in healthy tissues. Nanoparticles can be designed for the regulated and sustained delivery of therapeutic agents, such as miRNAs and natural chemicals, resulting in extended therapeutic efficacy and diminished dose frequency.^76^ They facilitate targeted delivery *via* surface modifications using ligands like folic acid or antibodies, which improve selectivity for osteosarcoma cells. Furthermore, nanoparticles can simultaneously deliver many therapeutic agents, including chemotherapeutic medicines, tumor-suppressing miRNAs, and natural anti-cancer compounds, resulting in synergistic benefits, mitigating drug resistance, and minimizing toxicity.^13,14,77^

Nanoparticles that integrate natural compounds and miRNAs provide a viable approach for osteosarcoma treatment. By merging the gene-regulatory capabilities of miRNAs with the therapeutic properties of bioactive natural substances, this methodology yields synergistic effects. miRNAs can inhibit oncogenic pathways, while natural chemicals deliver anti-tumor, anti-inflammatory, or sensitizing effects to traditional drugs.

### Metallic nanoparticles

Magnetic nanoparticles, particularly iron oxide-based systems, offer unique advantages for osteosarcoma therapy due to their dual functionality in targeted drug delivery and imaging-guided treatment. An investigation developed a multifunctional nanoplatform utilizing folic acid-conjugated Fe_2_O_3_ nanoparticles coated with polydopamine (PDA), a natural substance recognized for its biocompatibility and photothermal characteristics. This platform co-delivered miR-520a-3p and served as a photothermal treatment (PTT) agent. The method demonstrated synergistic anti-cancer effects through gene therapy and heat-induced tumor ablation, yielding superior outcomes compared to each modality alone.^14^

Another study utilized dextran, a natural polysaccharide, to encapsulate Fe_3_O_4_ nanoparticles, thereby creating a magnetic nanoparticle system with polyethylenimine (PEI) to deliver miR-302b. This technology inhibited osteosarcoma progression *in vitro* and *in vivo* through the regulation of the Hippo signaling pathway.^78^

### Polymeric nanoparticles

Polymeric nanoparticles represent one of the most extensively investigated platforms for miRNA and natural compound delivery due to their biodegradability, tunable physicochemical properties, and high loading capacity. An interesting study entails the co-delivery of miRNA-34a alongside the natural chemical resveratrol utilizing a chitosan and PLGA-based nanoparticle. This technique also incorporated doxorubicin for combinatorial therapy. The nanoparticle, constructed through a layer-by-layer application of pectin and chitosan, demonstrated pronounced tumor-suppressive effects in both 2D and 3D osteosarcoma models, markedly increasing apoptosis and impairing cellular function.^13^

A hydroxyl-rich, reduction-responsive polymer (TGIC-CA) was synthesized as a natural bioinspired biodegradable nanocarrier for miR-22 delivery. In combination with the PLK1 inhibitor volasertib, this co-delivery system significantly suppressed tumor proliferation and metastasis in patient-derived xenograft (PDX) models through TGIC-CA degradation in high-GSH tumor microenvironments, selectively releasing miR-22 to downregulate PI3K/Akt pathways.^79^

**Fig. 4:**
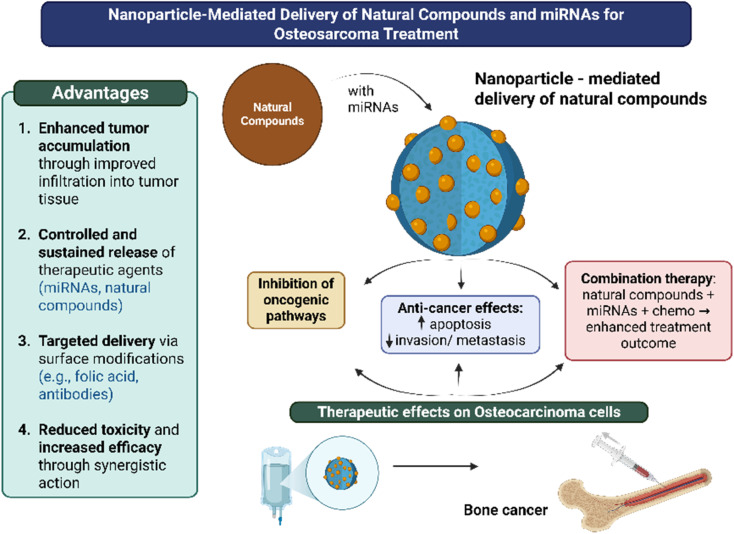
The nanoparticle-mediated delivery of natural compounds and miRNAs for the treatment of osteosarcoma.

Incorporating natural bioactive compounds, including resveratrol, polydopamine (PDA), folic acid, and dextran, into miRNA-delivery nanosystems has demonstrated significant promise in the therapeutic management of osteosarcoma. This combinatorial strategy leverages the intrinsic biological activities of these compounds, such as their antioxidant, anti-inflammatory, and bone-regenerative properties, alongside the targeted gene regulatory capacity of miRNA-loaded nanoparticles. The resulting synergistic effects contribute to enhanced apoptotic induction in tumor cells, effective tumor growth suppression, osteogenic differentiation promotion, and mitigation of systemic toxicity. Such multifunctional nanocarriers represent a novel and efficacious approach for targeted cancer therapy, offering improved therapeutic outcomes with reduced off-target effects in the treatment of osteosarcoma (Fig. 4).

In conclusion, Chitosan/PLGA nanoparticles co-delivering miR-34a, resveratrol, and doxorubicin have gone through preclinical testing, demonstrating tumor suppression in 2D/3D osteosarcoma models through increased apoptosis. Folic acid-Fe_3_O_4_-polydopamine (PDA) platforms for miR-520a-3p with photothermal therapy progressed to *in vivo* studies, demonstrating superior efficacy compared to single techniques in combined effect. Dextran-Fe_3_O_4_/PEI for miR-302b and TGIC-CA polymer for miR-22 (with volasertib) advanced to *in vivo* xenografts/PDX models, evaluating the improvements in targeted, GSH-responsive release compared to non-degradable systems. By improving bioavailability, these designs overcome earlier restrictions on free compounds.

### Comparative advantages and limitations of nano-delivered natural compounds *versus* current osteosarcoma therapies

The combination of nanotechnology and natural compounds is an important change aimed at overcoming the underlying limitations in the way osteosarcoma is currently managed. Here, we go over its possible benefits and intrinsic drawbacks in contrast to well-established clinical methods. Traditional chemotherapy frequently proves ineffective against metastatic, chemoresistant cells. The evaluated combinatorial nano-strategies address the metastatic and chemoresistant cells. For instance, nanoparticles that co-deliver resveratrol, miRNA-34a, and doxorubicin,^61,62^ might concurrently inhibit pro-survival pathways, cause apoptosis, and administer a cytotoxic medication, potentially overcome resistance to single agents. Likewise, geiparvarin-loaded nanoparticles aimed at miR-3912-3p/ANGPTL4 could selectively impede metastatic spread,^68^ a process inadequately managed by systemic chemotherapy. The dose-limiting cardiotoxicity of doxorubicin and the nephrotoxicity of cisplatin provide significant therapeutic challenges. Targeted nanocarriers, demonstrated by the folic acid-conjugated Fe_2_O_3_/PDA system,^14^ augment tumor-specific accumulation through active targeting and the enhanced permeability and retention (EPR) effect. This “passive and active” targeting can substantially reduce the necessary dosage of a potent natural substance (such as curcumin or ICS II) or co-administered chemotherapy, localizing the therapeutic action to the tumor microenvironment while maintaining healthy tissues. In contrast to single-target kinase inhibitors, some natural compounds as curcumin and ICS II intrinsically regulate multiple oncogenic pathways such as Wnt/β-catenin, Notch and miRNA clusters.^64,66^ Their previous utilization indicates predominantly positive safety characteristics. Nanotechnology addresses the principal limitations of poor bioavailability and quick metabolism by preserving the intended target and facilitating sustained, localized release, as evidenced by the dextran-coated Fe_3_O_4_ system for miR-302b delivery.^78^

Challenges encompass scalability for clinical production, the possible immunogenicity of polymers such as PEI in dextran-Fe_3_O_4_, and high-GSH-dependent degradation (TGIC-CA), which may differ among patients. The long-term stability and regulatory challenges of multifunctional nanoparticles are inferior to those of simpler formulations, and no human trials have been reported to yet.

### Challenges and future perspectives

Despite the promising preclinical results of combining natural medicines, miRNA modulation, and nanotechnology for osteosarcoma treatment, numerous significant difficulties must be resolved prior to clinical translation. A significant limitation is the absence of standardization of natural extracts, as variability in plant sources, extraction methods, and chemical compositions can profoundly influence bioactivity, reproducibility, and therapeutic efficacy, thus complicating validation studies and regulatory approval protocols.^80,81^ The scalability and manufacturability of advanced nanocarriers present significant challenges, as numerous multifunctional or multilayered nanoparticle systems are challenging to reproduce consistently under Good Manufacturing Practice (GMP) conditions, leading to concerns about cost, batch-to-batch variability, and long-term physicochemical stability.^82,83^ Moreover, while nanocarriers are generally designed for biocompatibility, their possible immunogenicity, unintended biodistribution, and prolonged accumulation in organs like the liver and spleen necessitate thorough toxicological assessment, especially for polymeric and magnetic nanoparticle-based gene delivery systems.^84,85^ Regulatory challenges constitute a substantial obstacle, as miRNA-loaded nanotherapeutics containing natural compounds are frequently categorized as intricate combination products, requiring thorough evaluation of pharmacokinetics, safety, manufacturing consistency, and therapeutic efficacy in the absence of fully harmonized regulatory frameworks for nanomedicine.^86,87^ Significantly, the majority of existing evidence endorsing these methodologies originates from *in vitro* studies or restricted animal models, which fail to adequately represent the heterogeneity, immune interactions, and metastatic characteristics of human osteosarcoma; thus, comprehensive *in vivo* validation utilizing orthotopic and patient-derived xenograft models, succeeded by meticulously designed early-phase clinical trials, is crucial to ascertain translational relevance and clinical viability.^2,3,79^

## Conclusion

The integration of natural compounds with nanotechnology presents a promising frontier in the development of targeted therapies for osteosarcoma. Natural bioactive substances, such as flavonoids, resveratrol, and other plant-derived extracts, possess inherent anti-inflammatory, antioxidant, and anti-tumor properties that can modulate critical molecular pathways involved in osteosarcoma progression. These compounds have demonstrated the ability to regulate miRNAs, which play pivotal roles in tumor growth, metastasis, apoptosis, and chemoresistance. By influencing miRNA expression, natural products can suppress oncogenic pathways and enhance tumor suppressor activity, ultimately inhibiting disease progression.

However, the effective delivery of these natural agents and miRNAs remains a significant challenge due to issues such as poor stability, low bioavailability, and non-specific targeting. Nanotechnology-based delivery systems, including layer-by-layer nanoparticles, magnetic nanocomposites, and biodegradable nanocarriers, address these limitations by enabling precise, controlled, and sustained release of therapeutic agents directly to tumor sites. Such nanocarriers can be decorated with targeting ligands like folic acid or antibodies to improve specificity for osteosarcoma cells, thereby reducing off-target effects and systemic toxicity. The co-delivery of natural compounds and miRNAs *via* nanocarriers harnesses the synergistic potential of these agents, amplifying anti-tumor effects and overcoming resistance mechanisms. This combinatorial approach not only enhances apoptosis and inhibits proliferation but also impedes metastasis and tumor recurrence. Importantly, these strategies contribute to a more personalized and effective therapeutic paradigm that leverages the biological activity of natural substances alongside advanced nanotechnologies.

## Author contributions

Conception and design: G. S. E., S. H. A., and A. S. D. collection and/or assembly of data: W. A. E., A. A, M. A., and H. H. M. manuscript writing: A. E. E., and O. A. M. All authors have read and approved the published version of the manuscript.

## Conflicts of interest

There are no conflicts to declare.

## Data Availability

No primary research results, software or code have been included and no new data were generated or analysed as part of this review.
